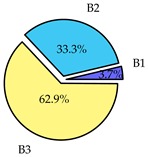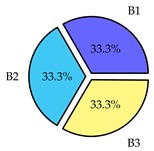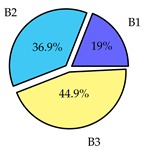# SAFE-MAC: Speed Aware Fairness Enabled MAC Protocol for Vehicular Ad-hoc Networks †

**DOI:** 10.3390/s19102405

**Published:** 2019-05-26

**Authors:** Md. Abubakar Siddik, Shafika Showkat Moni, Mohammad Shah Alam, William A. Johnson

**Affiliations:** 1 IICT, Bangladesh University of Engineering and Technology, Dhaka 1000, Bangladesh; siddik.ece@hstu.ac.bd; 2 Department of Computer Science, University of Kentucky, Lexington, KY 40506, USA; shafika.moni@uky.edu; 3 Department of Computer Science, Tennessee Technological University, Cookeville, TN 38505, USA; wajohnson43@students.tntech.edu

**Keywords:** Vehicular Ad-hoc Network (VANET), Vehicle to Infrastructure (V2I), MAC protocol, fairness, safety and non-safety applications

## Abstract

Highly dynamic geographical topology, two-direction mobility, and varying traffic density can lead to fairness issues in Vehicular Ad-hoc Networks (VANETs). The Medium Access Control (MAC) protocol plays a vital role in sharing the common wireless channel efficiently between vehicles in a VANET system. However, ensuring fairness between vehicles can be a challenge in designing MAC protocols for VANET systems. The existing protocol, IEEE 802.11 DCF, ensures that the packet transmission rate for a particular vehicle is directly proportional to the amount of time a vehicle spends within a service area, but it does not guarantee that faster vehicles will be able to send the minimum number of packets. Other existing MAC protocols based on IEEE 802.11 are able to provide a minimum amount of data transmission regardless of velocity, but are unable to provide an amount of data transmission that is more proportionate to the time a vehicle spends in the service area. To address the above limitations, we propose a Speed Aware Fairness Enabled MAC (SAFE-MAC) protocol that calculates the residence time of a vehicle in a service area by using mobility metrics such as position, direction, and speed to synthesize the transmission probability of each individual vehicle with respect to its residence time. This is achieved by dynamically altering the values of parameters such as minimum contention window, maximum backoff stage, and retransmission limit in the MAC protocol. We then develop an analytical model to compare the performance of our proposed protocol with contemporary MAC protocols. Numerical analysis results show that our proposed protocol significantly improves fairness among the speed-varying vehicles in VANET.

## 1. Introduction

Academia, transportation services, and the automotive industry have long awaited the realization of a completely autonomous VANET system that can prevent collisions, reduce wasteful driving, stay up to date with real time traffic information, and provide streaming entertainment to commuters. VANET are a subset of Mobile Ad-hoc Networks (MANETs) that offer a promising avenue towards these goals [1]. VANET is implemented via Vehicle to Vehicle (V2V), Vehicle to Infrastructure (V2I), and Vehicle to hand held Device (V2D) communications. These communications can be referred to collectively as Vehicle to Anything (V2X) communications [2]. In its early stages, VANET was only proposed for safety applications [3]. More recently however, VANET has been applied to more general features such as traffic safety, traffic information, and infotainment [4]. Safety applications include a variety of detection, warning, and avoidance mechanisms to prevent road accidents. Non-safety applications include passenger infotainment, driving assistance, and traffic efficiency and management systems [5,6,7,8]. The major requirements of safety applications are low latency and high reliability. High throughput is the major concern for non-safety applications.

Unique characteristics of VANET systems include highly dynamic geographic topology, predictable two-direction random mobility, strict delay constraints, varying node density, and rapid joining and leaving rate. While these characteristics distinguish VANET as a unique research field within MANET [4,5,9,10,11,12,13,14], they also impose several constraints onto MAC protocol design. Important goals in designing a MAC protocol for VANET are fairness, Quality of Service (QoS), reliability, time-bound delivery, robustness, distributed, and on demand channel access, among others [10]. Due to the use of a single radio channel, the MAC protocol is of paramount importance in VANET systems. The MAC protocol is responsible for sharing the common medium among the contending nodes. The primary goal of an efficient MAC protocol is to fairly share common resources such as bandwidth, and access time among participating nodes, while also maximizing throughput and minimizing access delays.

Due to the relative velocity between nodes (vehicle and Road Side Unit (RSU)), as well as different velocities of the vehicles within the service area of an RSU, the residence time of different vehicles can vary widely. This can adversely affect the chance of communication of a vehicle within the RSU. Vehicles may not be able to get the information that they want or need within an acceptable time without disturbing others. We refer to this as the fairness problem. With respect to only safety applications, all vehicles need a similar chance to transmit data. This requirement is defined by absolute fairness and is ensured by a modified IEEE 802.11-based MAC scheme [3]. To support safety and non-safety applications, high velocity vehicles will require a minimum chance to communicate to the RSU, such that they can at least complete the safety information. Other vehicles will also need chances proportional to their residence time so that they may communicate with the RSU effectively. This relative fairness is called proportional fairness [15,16].

The IEEE 802.11p standard [17] defines MAC and PHY layer specifications for VANET. It uses a MAC layer that is adopted from the IEEE 802.11e EDCA standard. The IEEE 802.11e EDCA provides service differentiation and QoS. In EDCA, the node separates its arrival traffic into four categories based on priority. Each category is called an Access Category (AC). Performance analysis of this protocol shows greater QoS than IEEE 802.11 DCF [18]. Service differentiation in IEEE 802.11p when providing QoS is not effective. This is because faster vehicles with high priority service may not be able to gain the data transmission, they need due to their low residence time. Instead, slower vehicles with lower priority may still easily communicate with the RSU. As a result, service prioritization is disturbed. Therefor in VANET, the residence time of vehicles has great impact on the achievement of QoS. Since the IEEE 802.11 DCF protocol has achieved some widespread popularity in WLANs, it seems to be a promising choice for V2I communications in VANET. In [19], V. Nguyen et al. discuss about different types of MAC protocols which are based on IEEE 802.11p. Their protocols use dynamic interval schemes to enhance throughput and reduce access delay.

The IEEE 802.11 standard MAC protocol [20] does not provide a minimum chance for high velocity vehicles to communicate with RSU, because the same MAC parameters are utilized by all vehicles. As a result, this protocol is not suitable for safety applications. The protocol provides a certain chance that is proportional to residence time for all vehicles. This makes it more applicable to non-safety applications. A modified IEEE 802.11 DCF based fair access MAC scheme is proposed in [3] that ensures absolute fairness by providing equal chance for all vehicles to communicate with the RSU. This protocol assumes that all vehicles will only transmit safety messages. As a result, absolute fairness is not afforded to both safety and non-safety applications.

To resolve this issue, we proposed a Speed Aware Fairness Enabled MAC (SAFE-MAC) protocol based on IEEE 802.11 DCF for V2I communications. Utilizing residence time, our protocol groups all vehicles into three batches. By considering their own position, direction, and velocity after a certain time interval, each vehicle calculates a batch number. Vehicles then use this batch number to adjust the MAC parameters to suit their respective batch. If a vehicle’s velocity changes, it can recalculate its batch number to suit its new velocity. This allows every vehicle the chance to enter into the high priority batch. Based on the analytical model, our protocol sets MAC parameters for each batch individually. This ensures that faster vehicles will be afforded some minimum number of messages, while message transmission rate of other vehicles is directly proportional to their residence time. As far as we are aware, there exists no other efficient MAC protocol that can provision proportional fairness in V2I communications while also realizing the requirements to support both safety and non-safety applications. A preliminary version of this work was presented in [21].

### 1.1. Contributions of the Paper

The main contributions of this paper, building on a complete description of MAC protocol for provisioning fairness, are summarized as follows:

- We propose and design a Speed Aware Fairness Enabled MAC (SAFE-MAC) protocol to ensure fairness in V2I communications.
- We introduce a new algorithm to calculate residence time of a vehicle in the service area of a RSU using direction, position, and speed of vehicles for both straight road and intersection road scenarios.
- We propose a new batch selection and update algorithm which uses instantaneous residence time of the vehicles to ensure proportional fairness.
- We develop an analytical model using 2-D Markov chain and derive some equations from this model for the proposed SAFE-MAC protocol under consideration for both saturated and non-saturated conditions of the vehicle queue.
- We evaluate our proposed protocol in terms of collision probability, channel idle probability, successful probability, packet drop probability, transmission probability, normalized throughput, and total normalized throughput while considering a dense network that resembles an urban scenario. We also investigate the effect of vehicle density on the total normalized throughput.
- Last but not least, we present a comprehensive view of the comparisons that describes the percentage of transmitted packets of different batches under different MAC protocols including the proposed SAFE-MAC protocol.

### 1.2. Organization of the Paper

The rest of the paper is organized as follows: Background of vehicular communications is discussed in Section 2. Problem statement is outlined in Section 2.3. In Section 3 brief discussion of previous work of fairness problem is presented. The proposed SAFE-MAC protocol that distributes fairness is described in Section 4. Section 5 derives the analytical model that carry out the performance analysis of the network solving fairness problem in a non-saturated state. Section 6 shows the numerical results of the model derived in Section 5. Finally, Section 7 concludes this paper.

## 2. Background

In this section, we review the architecture and the components of the network of vehicular communications. We also discuss our problem statement and the fairness issues in resource allocation.

### 2.1. VANET System Architecture and Components

**Figure 1:**
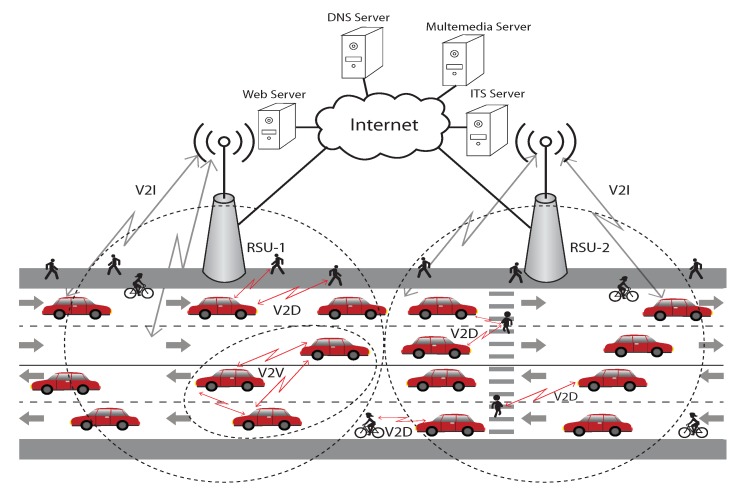
VANET architecture.

VANET utilizes two Service Sets (SS) toward network handling. The first is called the Basic Service Set (BSS) and is used in the communication between RSU and On Board Units (OBUs). The second is known as the Independent Basic Service Set (IBSS), and it supports communications between two nodes in a mesh network such as V2V communications. Figure 1 represents the RSU communication zones. The OBUs move between communication zones and exchange information with RSUs. To define different WLAN communication zones, each SS uses a unique identifier. Vehicles must associate with only one SS at a time. VANET specifies different types of DSR devices such as (a) OBUs with Global Positioning Systems (GPS) receivers located inside the vehicle (b) stationary RSU placed along the roadside, and (c) hand-held devices that are carried by pedestrians, cyclists, roadside workers, and/or driver passengers. RSUs are typically established in an elevated position on existing transport infrastructure i.e., traffic light, road sign, etc.

### 2.2. Fairness in Resource Allocation

Fairness is a broad concept in communication systems. MAC protocols are responsible for sharing common resources fairly among participating nodes. Unfair resource allocation among different individuals could cause resource waste and starvation as well as redundant resource allocation. It is also important to note what characterizes the fairness that we are interested in. Fairness can be seen from different perspective such as targeted or resultant fairness, short-term or long-term fairness, and system or individual fairness [22].

Another attribution of fairness is absolute or relative. Absolute fairness is defined as a situation in which each node has precisely the same amount of resources (time, throughput, etc) as every other node in the network. It is measured by either the Jain fairness indexed proposed by Jain et al. [23] or the entropy function introduced by Shannon [24]. Since different traffic types have different requirements based on their velocities, absolute fairness is not a very useful measure. Relative fairness is a better method to quantify fairness, because it accounts for however many of the individual requirements are taken into account. Overall relative fairness is then calculated by comparing how fulfilled the individual requirements are. There are two types of relative fairness: max–min fairness and proportional fairness. Max–min fairness is characterized by nodes with a smaller number of packets fulfilling all of their demand, while nodes with a larger number of packets having to share the remainder of the capacity equally [25]. Conversely, proportional fairness tries to keep a balance between maximizing network throughput while also allowing a minimum level of service to all nodes [15,16]. This criterion also favors nodes with a smaller number of packets, but it does not favor them as heavily as max-min fairness does.

### 2.3. Problem Statement

VANET can be uniquely characterized by highly dynamic geographic topology, predictable two-direction mobility, and varying vehicle density. All of these characteristics allow for a problem in fairness. The fairness problem can be defined as the case where higher velocity vehicles do not have enough chance to perform V2I communications or V2V communications with lower velocity vehicles. To allow for both safety and non-safety applications, we must ensure proportional fairness among the contending vehicles. In this paper, our goal is to design an efficient MAC protocol which can ensure proportional fairness of the networks.

## 3. Previous Works

The fairness issue with respect to faster vehicles has been explored for V2I scenarios [3,26,27,28,29,30,31] and for V2V scenarios in [32]. In [32], W. Alasmary et al. propose two dynamic CW-based mechanisms to reduce degradation of performance due to different velocities of vehicles. They do not, however, describe any processes to select an optimal contention window size to avoid the fairness problem. E. Karamad et al. propose a modified IEEE 802.11 DCF based MAC protocol in [3] that can dynamically assign a minimum contention window to vehicles of each batch by adjusting transmission probability with respect to speed. This minimum contention window is assigned at the beginning of access, and is utilized for the entire duration of access time. [3] ensures absolute fairness in the case that the network is in a state of saturation. It also derives relations between average velocity and minimum contention window for each batch by utilizing analytical approximations. The performance model for this model is based on the Bianchi model [33], and has the following assumptions:

1. Every node of the network always has at least one packet to transmit.
2. There are no hidden or exposed terminals in the network, and there is no capture effect.
3. Each packet collides with constant probability that is independent of the node state and other packets.
4. Transmission channel is ideal and transmission errors occur due to packet collision only.

In [26], Q. Wu et al. analyzed the performance of a MAC protocol proposed by E. Karamad et al. [3] in a saturated network state. [26] also derives the relationship between the minimum contention window size of a vehicle and the transmission probability, and the relationship between minimum contention window size of a vehicle in a network saturated state, and the average velocity. Both authors [3,26] assume the following concerning network model:

1. Vehicles arrive in the service area of an RSU in batches with respect to Poisson process with constant arrival rate.
2. The vehicles in each batch have the same average velocity which stays the same throughout the entire duration of the residence time of a particular RSU.
3. Vehicles do not change direction, and batch numbers stay fixed for each vehicle during the entire residence time.

Both authors [3,26] ensure the absolute fairness of the network, but they do not guarantee the proportional fairness of the network. This is appropriate for safety applications, but does not allow for non-safety applications.

In [27], V. P. Harigovindan et al. ensure equal opportunity for vehicles of different average speed in the service area of an RSU by deriving the analytical expression for optimum CW. They do not however analyze the performance of the network in both saturated and non-saturated states. Although this protocol can achieve absolute fairness, it does not consider the proportional fairness that we require for safety and non-safety applications.

To address the fairness problem of V2I communications, Hoeft et al. [28] proposed an algorithm to select RSU for OBUs. This algorithm minimizes the variation of OBUs connect to each RSU. However, they do not solve the fairness problem among vehicles with different velocities for a particular RSU. In [29], Zhang et al. build a Mixed Service Mobility (MSM) model for the IEEE 802.11p scheme to analyze the interactions between delay-tolerant services and real-time services under high speed mobility conditions. This contrasts heavily with the four different Access Categories defined by the IEEE 802.11p standard. While [29] achieves long term fairness for delay-tolerant services, they do not achieve fairness for real time services. In [30], S. A. Hussain et al. proposed a RSU-based efficient channel access scheme for VANETs under high traffic and mobility conditions. It dynamically adapts the contention window of each vehicle based on its deadline of departure from the range of RSU. The contention window for higher priority packets is varied slowly and vice versa for lower priority ones. However, their protocol ignores the case where changes may occur in Early Deadline First (EDF) (i.e., from low to high or high to low) due to a change in vehicle’s velocity or direction. It is also observed that a poor Jain’s fairness index is achieved for low to moderate traffic density. A velocity-adaptive V2I fair-access scheme based on IEEE 802.11 DCF for platooning vehicles is proposed in [31]. Their scheme achieves absolute fairness by varying Contention Window (CW) according to the velocity.

In this paper, we consider two important issues in designing an Speed Aware Fairness Enabled MAC (SAFE-MAC) protocol: high residence time variation among contending vehicles and vehicular communications for safety and non-safety applications. We believe that these issues can be solved by the applications of proportional fairness. To the best of our knowledge, we are the first to design an efficient MAC protocol for VANET systems to provision proportional fairness. In this way, we support both safety and non-safety applications.

## 4. Proposed Speed Aware Fairness Enabled MAC (SAFE-MAC) Protocol

In this section, the proposed SAFE-MAC is presented in detail. Assumptions and the system model of the proposed SAFE-MAC protocol are discussed.

### 4.1. System Model and Assumptions

**Figure 2:**
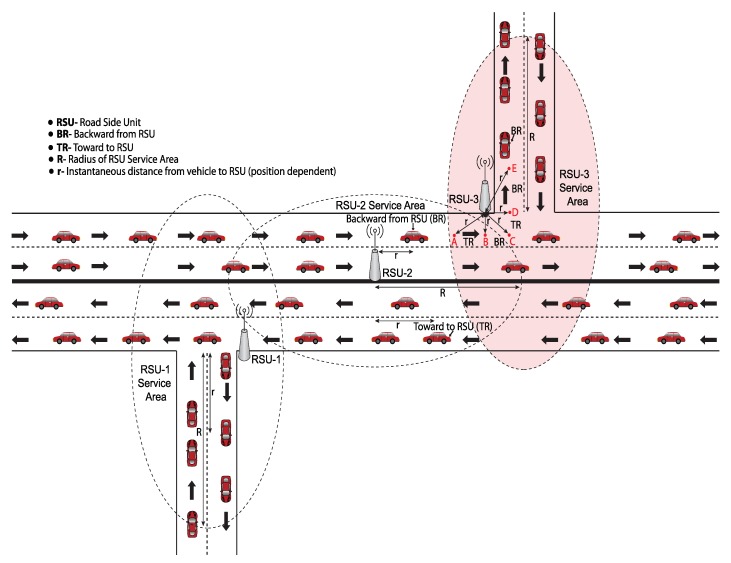
A V2I based VANET model.

In this paper, we consider a simple traffic model in which vehicles travel along a straight path with an intersection with bidirectional traffic covered by AP or RSU at a fixed position on either the road divider or along the road side as shown in Figure 2. The traffic model allows vehicles to change their direction and speed. Our network model groups vehicles into three classes based on their residence time.

1. Vehicles will arrive in an RSU service area according to a Poisson process and there will be no transmission error due to defective channels.
2. Vehicle to RSU links are symmetric and the effects of hidden terminals, exposed terminals, and channel capture are ignored.
3. Vehicles are able to measure their own positions, moving directions, and speeds.
4. Two non-overlapping channels are used by neighboring RSUs, and these channels will not interfere with each other.
5. Vehicles are equipped with IEEE 802.11 enabled communication devices.
6. All vehicles and RSUs have some unique identification number.
7. Within the service area of an RSU, the RSU and the vehicles operate at the same frequency.
8. Errors may occur because of packet collisions. We define a packet collision to be the case when two packets arrive at the same vehicle or RSU at the same time.

In assumption 2, the effects of hidden terminals, exposed terminals, and channel capture are ignored. These three dominant MAC layer issues are extensively studied in [34,35,36]. Overall throughput of the system is degraded for the presence of hidden terminal and exposed terminal in the network. Throughput is increased when capture effect is considered in the network. However, we do not consider hidden or exposed terminals because we try to find out the sole impact of MAC parameters on the performance of SAFE-MAC protocol. We also ignore the channel capture effect, a mechanism that auto-corrects some of the packet collisions, to allow us in measuring the actual packet collisions due to MAC parameter alone. The two contemporary protocols that we compared our proposed SAFE-MAC protocol with, also do not consider the hidden terminal, exposed terminal and capture effect.

### 4.2. Proposed SAFE-MAC Protocol

**Figure 3:**
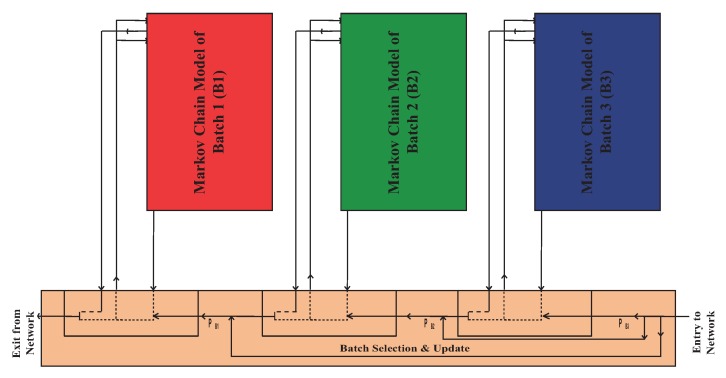
Channel access mechanism of proposed SAFE-MAC protocol.

The proposed SAFE-MAC protocol is a channel access mechanism specifically designed to avoid the fairness problem which is shown at the MAC layer. It is based on IEEE 802.11 DCF. It groups all the vehicles in one RSU service area into three batches. These batches are denoted as Batch 1, Batch 2, and Batch 3. Each batch’s vehicles are organized into a queue, and independently contend for transmission with the RSU. Requirements of safety and non-safety applications for VANET are extensively studied in [37]. Authors have discussed safety and non-safety applications separately because they used multi-channel MAC protocol based on IEEE 802.11p standard. Although multi-channel MAC protocol have higher performance than that of single-channel MAC protocols. We have considered a single-channel MAC protocol based on IEEE 802.11 because of low cost, less complexity, and popularity. IEEE 802.11 DCF do not consider service differentiation. In our proposed MAC, both safety and non-safety applications can be applicable. If both safety and non-safety messages are generated at the same time in one vehicle, and the channel is accessed successfully by that vehicle, safety application messages will be transmitted first followed by the non-safety messages. When vehicles enter the service area of an RSU, they perform the following steps to communicate with the RSU:

1. At first, every vehicle will enter into the service area of a particular RSU and associate with that RSU.
2. Vehicles will compute their moving direction, speed, and position within the network using a GPS receiver on their OBUs. Vehicles will then compute their residence time using Algorithm 1. Based on their residence time, vehicles will be associated as a member of Batch *i*. The detailed procedure of batch selection and batch update are given in Algorithm 2.
3. Every vehicle will listen to the channel to determine if it is idle. In the case that the time the channel sits idle reaches the DIFS time for a particular vehicle, that vehicle will enter the backoff procedure and execute steps from 5.
4. If the channel is perceived to be busy, the vehicle will do nothing and continue to listen to the channel for idleness. In the case that the time that the channel has been idle reaches the DIFS time for a particular vehicle, that vehicle will execute steps from 5.
5. Every vehicle will check the queue. If the vehicle has at least one packet in the queue, the backoff instant will enter the backoff procedure. If the vehicle has no packets to transmit, the backoff instant will enter into the post-backoff procedure.
6. In the post-backoff procedure, the backoff instant of the vehicles will start a backoff counter with an initial value randomly selected from $\left[0,W_{i,0}-1\right]$, where $W_{i,0}={CW}_{min(i)}$. After this, the vehicle will execute the steps from 8.
7. In the backoff procedure, the backoff instant of the vehicle will start a backoff counter with an initial value randomly selected from $\left[0,W_{i,0}-1\right]$, where $W_{i,0}={CW}_{min(i)}$. After this, the vehicle will execute the steps from 10.
8. During the post-backoff procedure, if the channel is perceived to be busy and the queue becomes empty, the backoff counter will stop at its current value. When the channel has become idle and stayed that way for DIFS time, the backoff counter will resume. If the channel is perceived to be idle in a time slot (σ) and the queue becomes empty, the backoff counter will be decremented by one. When the backoff counter reaches zero, the queue will wait to receive a packet, and wait a predefined time interval. After this, the vehicles will execute from 2.
9. During the post-backoff procedure, if the channel is perceived to be busy and the queue has at least one packet to transmit, the backoff instance moves to the backoff procedure without changing the backoff counter. If the channel is perceived to be idle and the queue has at least one packet to transmit, the backoff instant moves to the backoff procedure and the backoff counter will be decremented by one. After that, the vehicle continues to the next steps.
10. During the backoff procedure, if the channel is perceived to be busy, the backoff counter will stop at its current value and the vehicle will continue to listen on the channel until the channel has been idle for up to the DIFS time. After this, the backoff counter will resume. Then If the channel is perceived to be idle in a time slot (σ), the backoff counter will be decremented by one. When the backoff counter reaches 0, the packet will be transmitted.
11. If the transmission is successful, the vehicle executes the steps from 2.
12. If the vehicle is not successful in sending its packet, the packet will be retransmitted. At the end of the retransmission, the backoff instant of the vehicle will start a new backoff counter, setting its value randomly from $\left[0,W_{i,j}-1\right]$, where $W_{i,j}={2^{j}\times CW}_{min(i)}$ and *j* is the number of retransmission. After this, the vehicle executes the steps from 2.
13. If the value of $W_{i,j}$ reaches $CW_{max(i)}$, the backoff instant keeps the contention window size ${CW}_{max(i)}$. Then the vehicle will try to retransmit up to the retransmission limit ($m_{i}+x_{i}$) without changing the contention window size using step 10. After the ($m_{i}+x_{i}+1$) times unsuccessful transmissions, the packet will be dropped and the vehicle will execute steps from 2.
14. When the residence time of the vehicle becomes zero, the vehicle will exit from the service area of the RSU and continue the above procedure under the next RSU. The channel access mechanism with batch update procedure is shown in Figure 3.

**Algorithm 1** Residence time calculation
1:when vehicle complete the association process with an particular RSU2:set radius of RSU = *R*3:compute the distance of vehicle from RSU = *r*4:determine the average velocity of vehicle = *v*5:determine the moving direction of the vehicle6:**if***r* < *R* **then**7:  **if** (direction is toward) **then**8:    residence time, $T_{r}=\;\frac{R\;+\;r}{v}$9:  **else**10:    residence time, $T_{r}=\;\frac{R\;-\;r}{v}$11:  **end if**12:
**else**
13:  go to the step 114:
**end if**



**Algorithm 2** Batch selection and update
1:calculate the instantaneous residence time $T_{r}$ using algorithm 12:set theoretical minimum velocity of vehicle = $v_{min}$3:set theoretical maximum velocity of vehicle = $v_{max}$4:calculate minimum residence time $T_{r(min)}$, maximum residence time $T_{r(max)}$ and intermediate residence time $T_{r(in)}$ using Equations (2)–(4) respectively5:**if** (0 < $T_{r}$ ≤ $T_{r(min)}$) **then**6:  batch number = 17:**else if** ($T_{r(min)}$ < $T_{r}$ ≤ $T_{r(in)}$) **then**8:  batch number = 29:**else if** ($T_{r(in)}$ < $T_{r}$ ≤ $T_{r(max)}$) **then**10:  batch number = 311:
**else**
12:  go to the step 113:
**end if**
14:set the MAC parameters of selected batch in DCF function15:attempt to transmit packet or wait for packet16:**if** (successful transmission **OR** packet drop **OR** waiting time cross a predefined time with empty queue) **then**17:  execute the step from 1 to step 1318:  **break**19:
**end if**
20:**if** (batch number changed) **then**21:  update the MAC parameters22:
**else**
23:  unchanged the MAC parameters24:
**end if**
25:go to the step 15


## 5. Modeling of the Proposed SAFE-MAC Protocol

In this section, we present an analytical model of the proposed SAFE-MAC using 2-D Markov chain. Batch selection, transmission probability determination for each batches and normalized throughput are discussed.

### 5.1. Batch Selection

In this section, we describe the batch selection and transmission probability determination of each batch to ensure proportional fairness. The network model is described in Figure 2, and the batch selection and update algorithm is shown in Algorithm 2. The instantaneous residence time, $T_{r}$, of a vehicle can be expressed as:

(1) $$T_{r}=\;\frac{R\pm r}{v}$$

where *R* is the radius of the service area of an RSU, *r* is the instantaneous distance from a vehicle to the RSU, *v* is the instantaneous velocity of the vehicle, ‘+’ indicates that the vehicle is moving toward to RSU and the—denotes that the vehicle is moving backward from RSU.

For the intersection scenario shown in Figure 2, the instantaneous residence time of a vehicles is also calculated by Equation (1). We consider an intersection point in the road which is marked by a color and covered by RSU. Consider the case that a vehicle is in the overlapping service area of RSU-2 and RSU-3. The vehicle will associate with RSU-3 due to strong carrier signal of RSU-3 compared to the carrier signal of RSU-2. Measurement of vehicle direction at the intersection is a very important issue. To understand this issue, we consider five marked positions which are denoted by **A**, **B**, **C**, **D** and **E**. If the vehicle moves from point **A** to point **E**, the direction of the vehicles from **A** to **B** and from **C** to **D** is toward the RSU. In this case, ‘+’ sign is used. For from **B** to **C** and from **D** to **E**, we use − sign because the direction is away from RSU. When the vehicle moves from point **E** to point **A**, the sign will be opposite in Equation (1) for these corresponding points.

The velocity of the vehicle is uniformly distributed in $\left[v_{min},v_{max}\right]ms^{-1}$. Different residence times of the vehicles are computed as:

(2) $$T_{r(min)}=\;\frac{2R}{v_{max}}$$

(3) $$T_{r(max)}=\;\frac{2R}{v_{min}}$$

(4) $$T_{r(in)}=\frac{T_{r(min)}+T_{r(max)}}{2}$$

where $T_{r(min)}$, $T_{r(in)}$ and $T_{r(max)}$ are minimum residence time, intermediate residence time, and maximum residence time respectively. Batch number selection of each vehicle is carried out on Table 1.

According to E. Karamad et al. [3], the packet transmission rate, $R_{i}$ of a vehicle in batch i(i=1,2,3) can be expressed as:

(5) $$R_{i}=H\times \frac{R_{bit}}{N_{bit}}\times \frac{P_{t(i)}}{\sum_{k=1}^{3}P_{t(k)}}$$

where *H* is the normalized throughput of the network, $N_{bit}$ is the average number of bits in a packet, $R_{bit}$ is the bit rate over the channel, and $P_{t(i)}$ is the transmission probability of the vehicle in Batch *i*. If the minimum number of packets transmitted by each vehicle to achieve proportional fairness is $N_{P}$ then the transmission probabilities of different batches will satisfy the following condition. Transmission probability of each batch can be calculated by using Equations (5) and (6):

(6) $$R_{i}\times T_{r(i)}=N_{P}$$

### 5.2. Markov Chain Analysis

Performance modeling of IEEE 802.11, IEEE 802.11e, and IEEE 802.11p standards have recently been studied [33,38,39,40,41]. In this paper, we develop an analytical model to measure the performance of our proposed SAFE-MAC protocol by assuming transmission error can only be caused by data collision. The summary of the used notations are listed in Table 2.

**Figure 4:**
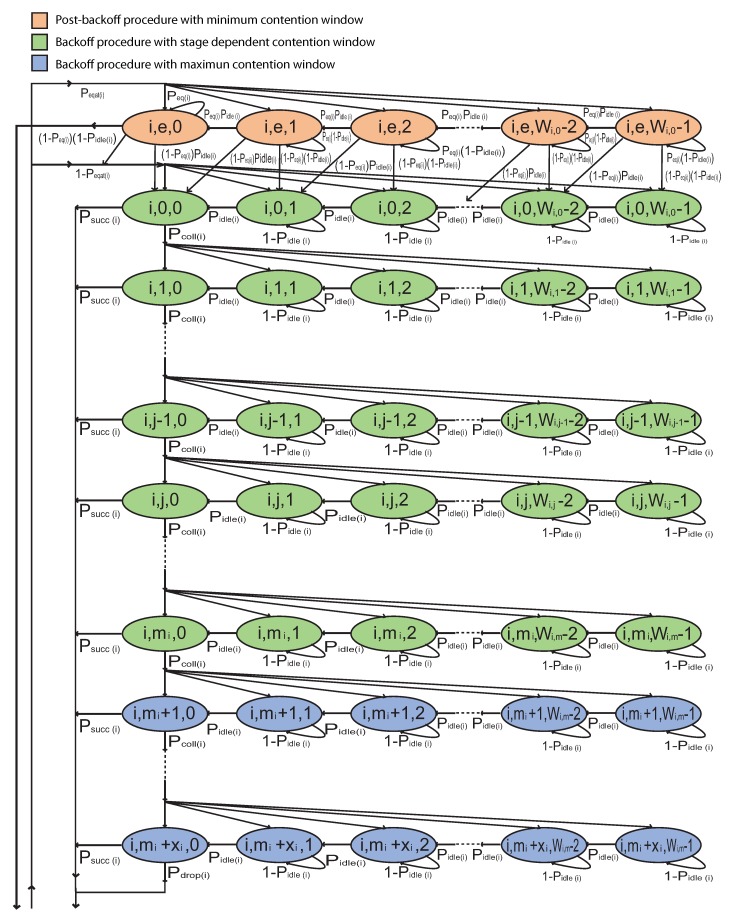
Markov chain model for the backoff procedure of a vehicle with both saturated and non-saturated state.

Firstly, we create a 2-D Markov chain to describe the backoff and post-backoff procedure of a vehicle queue for both non-saturated and saturated network states respectively. This can be seen in Figure 4. In this Markov chain, the state of each vehicle queue is denoted by (i,j,k) where i is an index denoted the batch number, *j* is the backoff stage number, and *k* is the backoff counter. The backoff stage *j* initializes at zero, and is incremented by one each time a packet is retransmitted until it reaches the retransmission limit $\left(m_{i}+x_{i}\right)$. After a successful transmission or packet drop, *j* is set to either 0 or *e* to represent either a non-empty or empty queue respectively. The value of *k* is initially set to a value uniformly selected from $\left[0,W_{i,j}-1\right]$ once the state reaches stage *j* and *k* is either decremented by one if the channel is perceived to be idle in a slot, or *k* is frozen if a transmission is detected on the channel. The backoff counter *k* is reactivated when the channel is perceived to be idle again for more than DIFS time. Transmission is attempted when the channel is perceived to be idle after *k* reaches 0. The backoff instant initiates the backoff procedure when the queue is not empty and post-backoff procedure when the queue is empty. The contention window of a vehicle of Batch *i* at stage *j* is defined as:

(7) $$W_{i,j}=\left\{\begin{array}{c} {CW}_{min(i)}\;\;;\;\;j=0\;or\;e\\ {2^{j}\times CW}_{min(i)}\;;\;\;1\leq j\leq m_{i}\\ {CW}_{max(i)}\;\;;\;\;m_{i}<j\leq m_{i}+x_{i} \end{array}\right.$$

At the time of backoff procedure, if the channel is sensed busy, the backoff counter is frozen to the present backoff value and if the channel is sensed idle, the backoff counter is decremented by one. For $0<k\leq W_{i,j}-1$, these one-step transition probabilities are given by:

(8) $$P_{i,j,k\;|\;i,j,k}=\;1-P_{idle(i)}\;;\;0\leq j\leq m_{i}+x_{i}$$

(9) $$P_{i,j,k-1\;|\;i,j,k}=\;P_{idle(i)}\;;\;0\leq j\leq m_{i}+x_{i}$$

During the backoff procedure, after each unsuccessful transmission attempt, the backoff instance moves down to below row at probability $P_{coll(i)}$ until reaches to maximum retransmission limit $m_{i}+x_{i}$ and chose a random backoff time at probability $1/W_{i,j}$. For $0\leq k\leq W_{i,j}-1$ and $\;\;0\leq j\leq m_{i}+x_{i}-1,$ this one-step transition probability is given by:

(10) $$P_{i,j+1,k\;|\;i,j,0}=\;\frac{P_{coll\left(i\right)}}{W_{i,j}}$$

After exceeding the retransmission limit, the packet is dropped at probability $P_{drop(i)}$.

(11) $$P_{drop(i)}=P_{coll(i)}^{m_{i}+x_{i}+1}$$

During the backoff procedure, after each successful transmission or packet drop, the backoff instance moves to second row at probability $P_{succ(i)}$ or $P_{drop(i)}$ respectively if there is a packet waiting in the transmission queue represented by probability $1-P_{eqat\left(i\right)}$ or moves of first row if there is no packet waiting in the transmission queue represented by the probability $P_{eqat(i)}$ and backoff instance enters to the post-backoff procedure. When backoff instance reaches to the state $\left(i,j,0\right)$ of Markov chain, the channel idle probability is zero. So that the probability of successful transmission is $1-P_{coll\left(i\right)}$. For $0\leq k\;\leq W_{i,0}-\;1$, these one-step transition probabilities are given by:

(12) $$P_{i,e,k|\;i,j,0}=\;\frac{\left(1-P_{coll\left(i\right)}\right)P_{eqat(i)}}{W_{i,0}}\;;\;0\leq j\leq m_{i}+x_{i}-1$$

(13) $$P_{i,0,k|\;i,j,0}=\;\frac{\left(1-P_{coll\left(i\right)}\right)\left(1-P_{eqat\left(i\right)}\right)}{W_{i,0}}\;;\;0\leq j\leq m_{i}+x_{i}-1$$

(14) $$P_{i,e,k|\;{i,m}_{i}+x_{i},0}=\;\;\frac{P_{eqat(i)}}{W_{i,0}}$$

(15) $$P_{i,0,k|\;{i,m}_{i}+x_{i},0}=\;\frac{\left(1-P_{eqat\left(i\right)}\right)}{W_{i,0}}$$

During the post-backoff procedure, if the channel is sensed idle and the queue is empty, the backoff counter is decremented by one, if the channel is sensed idle and the queue has at least one packet to transmit, the backoff counter is decremented by one and backoff instance jump to next backoff stage, if the channel is sensed busy and the queue is empty, the backoff counter is frozen, or if the channel is sensed busy and the queue has at least one packet to transmit, the backoff counter is frozen and backoff instance jump to next backoff stage. For $0<k\leq W_{i,0}-1$, these one-step transition probabilities are given by:

(16) $$P_{i,e,k|\;i,e,k}=P_{eq(i)}\left(1-P_{idle\left(i\right)}\right)$$

(17) $$P_{i,e,k-1\;|\;i,e,k}=\;P_{eq(i)}P_{idle(i)}$$

(18) $$P_{i,0,k-1\;|\;i,e,k}=\;\;\left(1-P_{eq\left(i\right)}\right)P_{idle(i)}$$

(19) $$P_{i,0,k|\;i,e,k}=\left(1-P_{eq\left(i\right)}\right)\left(1-P_{idle\left(i\right)}\right)$$

When backoff instance complete the post backoff procedure then it is waiting for a packet at probability $P_{eq(i)}$. If it receives a packet and channel is busy, the backoff instance shift down to next backoff stage but when channel is sensed idle and received a packet, the backoff instance moves to state (i,0,0) to attempt transmission.

(20) $$P_{i,e,0\;|\;i,e,0}=\;P_{eq(i)}$$

(21) $$P_{i,0,0\;|\;i,e,0}=\;\left(1-P_{eq\left(i\right)}\right)P_{idle(i)}$$

(22) $$P_{i,0,k|\;i,e,0}=\;\frac{\left(1-P_{eq\left(i\right)}\right)\left(1-P_{idle\left(i\right)}\right)}{W_{i,0}}\;;\;0\leq k\leq W_{i,0}-1$$

To compress the stationary probability equations, let $a=\sum_{j=0}^{m_{i}+x_{i}-1}P_{succ\left(i\right)}b_{i,j,0}+\;b_{{i,m}_{i}+x_{i},0}$ and $b=a\left(1-P_{eqat(i)}\right)+\left(1-P_{idle\left(i\right)}\right)\left(1-P_{eq(i)}\right)b_{i,e,0}$.

According to the 2-D Markov chain in Figure 4, all birth-death equations write recursively through the chain from upper row to lower row and from right to left, then the following stationary probabilities are given as follows:

(23) $$b_{i,e,0}=\frac{aP_{eqat(i)}}{W_{i,0}\left(1-P_{eq\left(i\right)}\right)}\frac{1-P_{eq(i)}^{W_{i,0}}}{1-P_{eq(i)}}$$

(24) $$b_{i,e,k}=\frac{aP_{eqat(i)}}{W_{i,0}P_{idle(i)}}\frac{1-P_{eq(i)}^{W_{i,0}-k}}{1-P_{eq(i)}}\;;\;0<k\leq W_{i,0}-1$$

(25) $$\begin{array}{cc} b_{i,0,k}= & \frac{\left(W_{i,0}-k\right)}{W_{i,0}P_{idle(i)}}b_{i,0,0}+\left(1-P_{eq(i)}\right)P_{idle(i)}b_{i,e,k+1}\\ & +\left(1-P_{eq(i)}\right)\left(1-P_{idle(i)}\right)b_{i,e,k}\;;\;0<k\leq W_{i,0}-1 \end{array}$$

(26) $$b_{i,j,o}=P_{coll(i)}^{j}b_{i,0,0}\;;\;0\leq j\leq m_{i}+x_{i}$$

(27) $$b_{i,j,k}=\frac{W_{i,j}-k}{W_{i,j}P_{idle(i)}}P_{coll(i)}^{j}b_{i,0,0}\;;\;0<j\leq m_{i}+x_{i}\;and\;0<k\leq W_{i,0}-1$$

By using Equations (23)–(27), all stationary probabilities $b_{i,j,k}$ are expressed by $b_{i,0,0}$ and which is finally determined by imposing the normalization condition, as follows:

(28) $$\sum_{k=0}^{W_{i,0}-1}b_{i,e,k}+\sum_{j=0}^{m_{i}+x_{i}}\sum_{k=0}^{W_{i,j}-1}b_{i,j,k}=1$$

$${\Rightarrow b}_{i,e,0}+\sum_{k=1}^{W_{i,0}-1}b_{i,e,k}+b_{i,0,0}+\;\sum_{j=1}^{m_{i}}b_{i.j.0}+\sum_{k=1}^{W_{i,0}-1}b_{i,0,k}+\sum_{j=1}^{m_{i}+x_{i}}\sum_{k=1}^{W_{i,j}-1}b_{i,j,k}=1$$

(29) $$\begin{array}{cc} \Rightarrow \frac{1}{b_{i,0,0}}= & \;1\;+\;\frac{\left({1-P}_{eqat\left(i\right)}\right)\left(W_{i,0}-1\right)}{2P_{idle\left(i\right)}}+\sum_{j=1}^{m_{i}+x_{i}}P_{coll\left(i\right)}^{j}\left(1+\frac{W_{i,j}-1}{2P_{idle\left(i\right)}}\right)+\\ & \frac{P_{eqat\left(i\right)}}{W_{i,0}}\left[\frac{1-{P_{eq\left(i\right)}}^{W_{i,0}}}{1-P_{eq\left(i\right)}}\left\{\frac{1-P_{idle\left(i\right)}}{2P_{idle\left(i\right)}}\left(\frac{2P_{eq\left(i\right)}}{1-P_{eq\left(i\right)}}\;+\left(W_{i,0}-1\right)\right)-\frac{1}{P_{eq\left(i\right)}}\right\}\right]\\ & +\;\frac{P_{eqat\left(i\right)}}{W_{i,0}}\left\{\frac{2\left(2-P_{eq\left(i\right)}\right)}{P_{idle}\left(1-P_{eq\left(i\right)}\right)}\;+\;\frac{1-P_{eq\left(i\right)}}{P_{eq\left(i\right)}}\right\} \end{array}$$

Packet transmission occurs if backoff counter becomes zero, regardless of the backoff stage. So packet transmission probability of a vehicle in a random time slot express as:

(30) $$P_{t(i)}=\;b_{i,0,0}\frac{1-P_{coll(i)}^{m_{i}+x_{i}+1}}{1-P_{coll(i)}}$$

Substituting Equations (29) and (30), the packet transmission probability of a vehicle in a randomly chosen time slot is produced.

### 5.3. Normalized Throughput Analysis

As can be seen from Equation (30), $P_{t(i)}$ is directly dependent on the conditional collision probability and the channel idle probability, which is still unknown. A vehicle of Batch *i* determines that the channel is idle if there are no other vehicles in Batch *i*, even if another batch transmits simultaneously in the same channel. A successful transmission can be defined as the case where only one vehicle transmits a packet in a particular time slot. A collision occurs when more than one vehicle transmits a packet in the same time slot. According to Y. H. Bae et al. [41], $\;P_{idle(i)}$, $P_{succ(i)}$ and $P_{coll(i)}$ can be calculated as follows:

(31) $$P_{idle(i)}=\frac{\prod_{k=1}^{3}{\left({1-P}_{t(k)}\right)}^{n_{k}}}{1-P_{t(i)}}$$

(32) $$P_{succ\left(i\right)}=\sum_{i=1}^{3}\frac{n_{i}P_{t\left(i\right)}}{{1-P}_{t\left(i\right)}}\prod_{k=1}^{3}{\left({1-P}_{t\left(i\right)}\right)}^{n_{i}}$$

(33) $$P_{coll(i)}=1-\sum_{i=1}^{3}\frac{n_{i}P_{t\left(i\right)}}{{1-P}_{t\left(i\right)}}\prod_{k=1}^{3}{\left({1-P}_{t\left(i\right)}\right)}^{n_{i}}-\frac{\prod_{k=1}^{3}{\left({1-P}_{t(k)}\right)}^{n_{k}}}{1-P_{t(i)}}$$

According to Bianchi model [33], the normalized throughput of Batch *i* of the network can be calculated as:

(34) $$S_{i}=\;\frac{{T_{p}P}_{succ(i)}}{T_{succ}P_{succ(i)}+\;T_{coll}P_{coll(i)}+\sigma P_{idle(i)}}$$

where $T_{p}$ is the average packet length (measured in time) σ is the time duration of an empty CSMA slot, $T_{coll}$ is the average duration of a packet collision, and $T_{succ}$ is the average duration of a successful packet transmission. Time intervals $T_{succ}$ and $T_{coll}$ depend on the access method used.

## 6. Analytical Results

In this section, we discuss the performance analysis of the proposed SAFE-MAC protocol based on the analytical model of Section 5. For simplicity, we assume that the packet size is 216 bytes, the probability that a packet is in the queue is 0.01, and the vehicle uses the basic access mechanism. The parameters used in our analysis are listed in Table 3.

**Figure 5:**
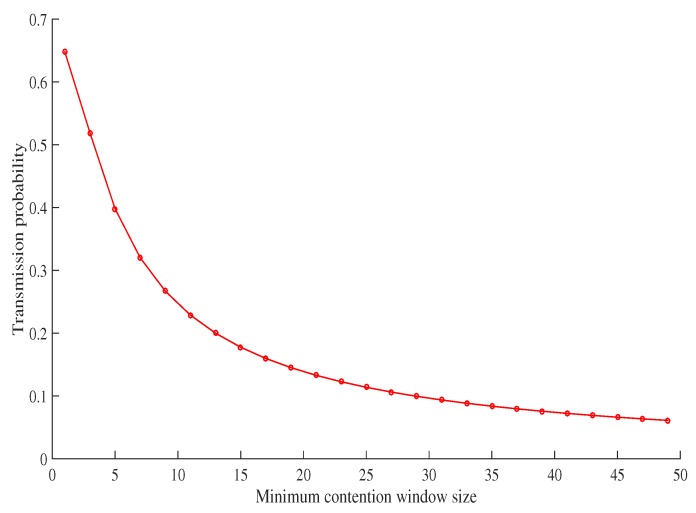
Selection of minimum contention window size based on transmission probability [21].

Figure 5 shows the relationship between the minimum contention size and the transmission probability of a vehicle. It can be seen that the two have an inverse relationship; as the contention size decreases, the transmission probability of a vehicle increases. To ensure fairness, the transmission probability of each batch is calculated by using Equations (5) and (6). The minimum contention window size of each batch seen in Figure 5, is determined using the calculated transmission probability. The maximum backoff stage and retransmission limit of each batch is a set, fixed value for the sake of simplicity. The MAC parameters can be seen in Table 4.

**Figure 6:**
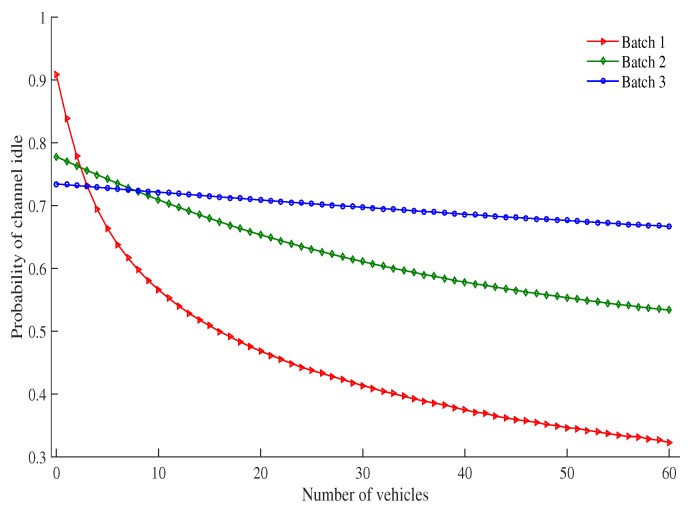
Probability of channel idle vs. number of vehicles.

Figure 6 shows the probability that a channel is idle for each batch with a different number of vehicles under the RSU service area. The probability that the channel is idle decreases as the number of vehicles increases. This is due to the fact that as the number of vehicles increases, there are more vehicles to contend with each other for transmission in the same time slot. This results in a decreased probability that the channel is idle. When the number of vehicles increases, the channel idle probability of the high priority batch decreases faster than other batches. For example, when the number of vehicles is 5, the channel idle probability of Batch 1, Batch 2 and Batch 3 are 0.66, 0.74 and 0.72 respectively. As the number of vehicles is increased to 50, the channel idle probability of Batch 1, Batch 2 and Batch 3 are 0.34, 0.55 and 0.67 respectively. reduction of channel idle probability in percentage is 48.48% for Batch 1, 25.67% for Batch 2 and 6.94% for Batch 3. Due to the low contention window size of the high priority batch, as compared with other batches, the probability of the number of contending vehicles in a slot is high for the same number of vehicles.

**Figure 7:**
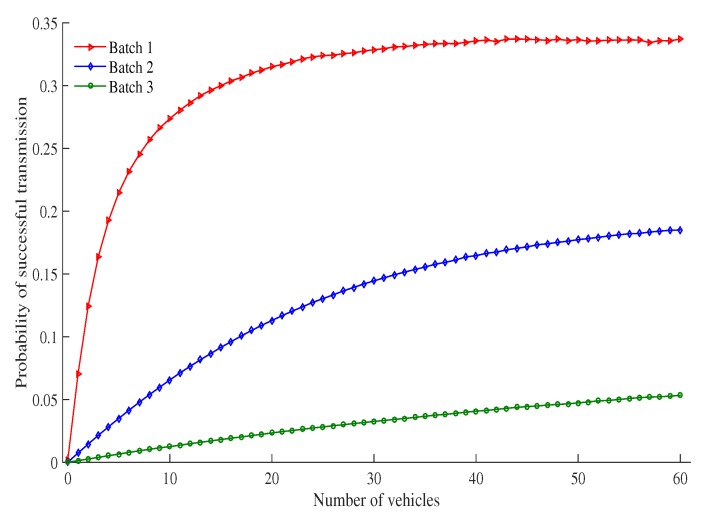
Probability of successful transmission vs number of vehicles.

Figure 7 shows the probability of a successful transmission as the number of vehicles under an RSU service area changes. The probability that the transmission is successful increases as the number of vehicles increases. This is due to the fact that as the number of vehicles increases, there are more vehicles to contend with each other for transmission in the same time slot. As a result, more collision occur as do successful transmissions. When the number of vehicles increases, the successful transmission probability of high priority batches increases faster than other batches and fixed in a saturated value. For example, probability of a successful transmission of Batch 1 is 0.21, 0.331 and 0.336 at the number of vehicles 5, 30 and 50, respectively. Due to low contention window size of high priority batch (as compared with other batches) the probability of the number of contending vehicles in a slot is high for the same number of vehicles.

**Figure 8:**
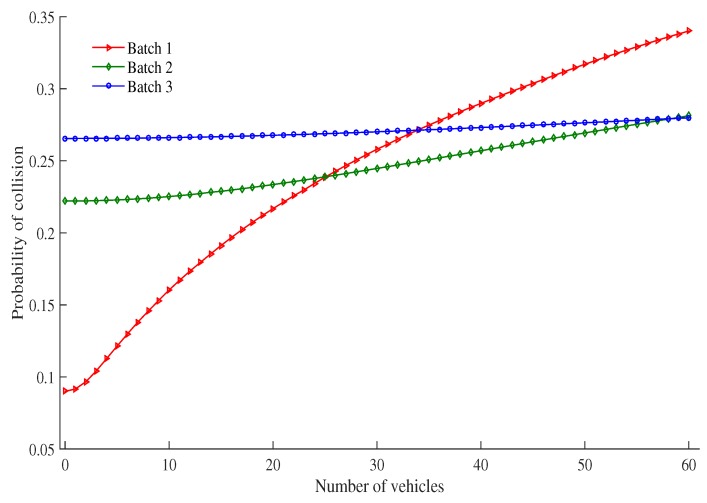
Probability of collision vs. number of vehicles.

Figure 8 shows the probability of collision as the number of vehicles under an RSU service area changes. The probability of collision increases as the number of vehicles increases. This is due to the fact that as the number of vehicles increases, there are more vehicles to contend with each other for transmission in the same time slot. It is also observed that the probability of collision of high priority batch vehicle is higher than other batches when the density of vehicles is increased, especially when the number of vehicles is greater than or equal to 33. After 33, the channel idle probability is very low (0.34 for 50 vehicles) and successful probability tends to a saturated value (0.33), so that collision probability of high priority batch vehicle is higher than other batches. It is calculated by Equation (33).

**Figure 9:**
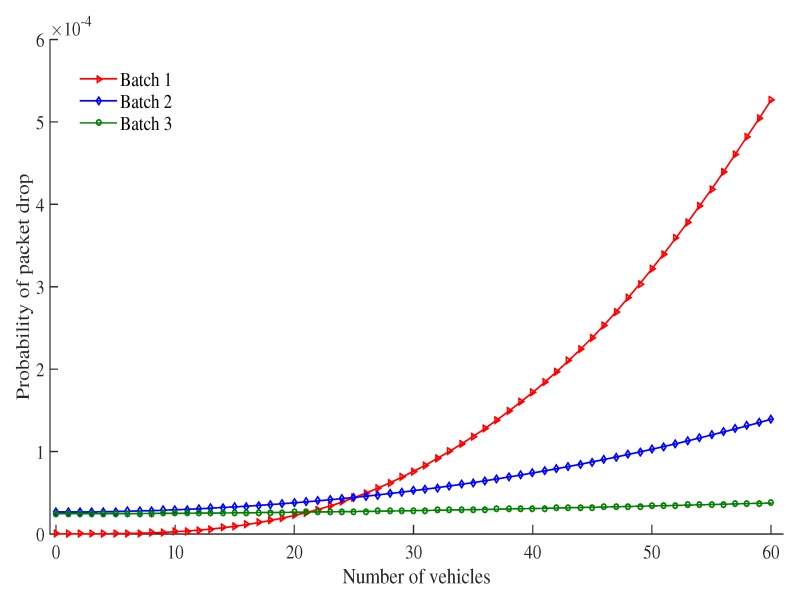
Probability of packet drop vs. number of vehicles.

Figure 9 shows probability of packet drop as the number of vehicles under an RSU service area changes. The probability that the packet is dropped increases as the number of vehicles increases. This is due to the fact that as the number of vehicles increases, there are more vehicles to contend with each other for transmission in the same time slot and more collision occur. As a result more packets are dropped. When the number of vehicles increases, the probability of packet drop of high priority batch increases faster as compared to other batches. For example, when the number of vehicles are increased from 30 to 60, the probability of packet drop of Batch 1, Batch 2 and Batch 3 are also increased 7.0, 2.65 and 1.35 times higher than previous. Due to low contention window size of high priority batch (as compared with other batches), the probability of number of contending vehicles in a slot is high for the same number of vehicles. The results are more collision in tge high priority batch and more packets are dropped.

**Figure 10:**
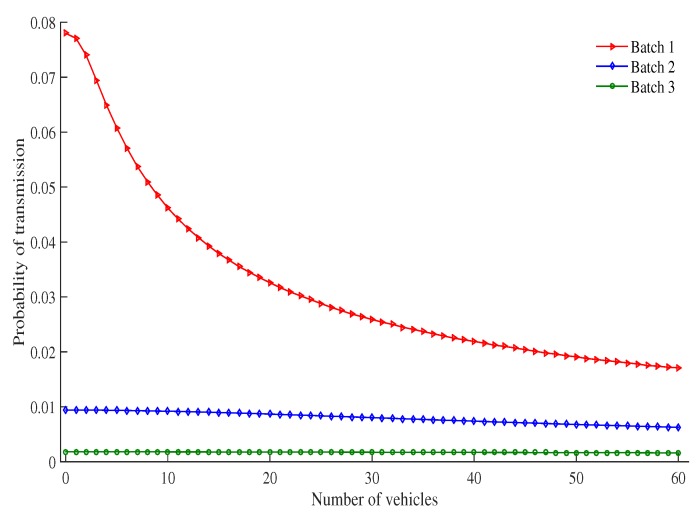
Probability of transmission vs. number of vehicles.

Figure 10 shows the probability of transmission for each batch of vehicles as the number of vehicles increases under a particular RSU service area. The probability of transmission decreases as the number of vehicles increases. This is again due to the fact that as more vehicles are introduced, more vehicles contend with each other to transmit messages in a certain time slot. This causes more vehicles to stay in the backoff stage for longer, which results in an increased probability of collision and an increase of time needed to access the channel. This leads to an overall decrease in transmission probability.

**Figure 11:**
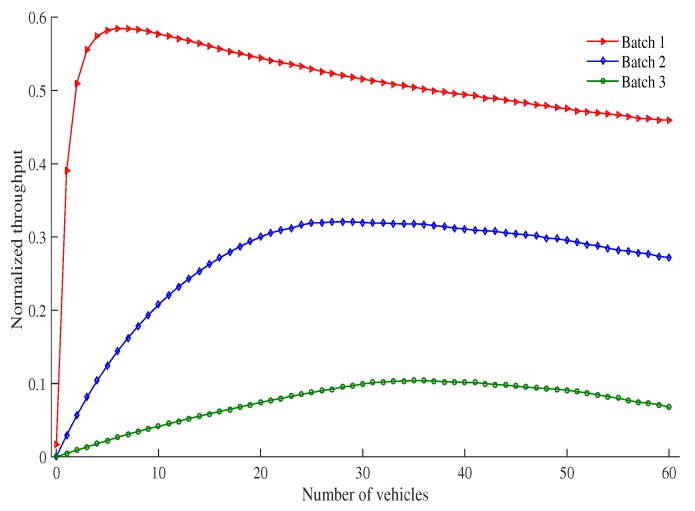
Normalized throughput vs. number of vehicles.

Figure 11 shows the normalized throughput of each batch of vehicles as the number of vehicles in an RSU service area changes. It can be seen that the normalized throughput of each batch approaches a maximum value as the number of vehicles increases. This is due to the fact that a small number of vehicles leads to a low normalized throughput for every batch. As the number of vehicles increases within a range, it would not result in many collisions. This results in an increase in the normalized throughput for each batch. As the number of vehicles continues to increase, the network enters into a saturated state. In such a state, more vehicles will be contending for transmission at any given time, which results in an increase in collisions, and therefor a reduction in the normalized throughput for all batches. For example, maximum normalized throughput of Batch 1, Batch 2, and Batch 3 are achieved when the number of vehicles are 6, 28, and 35, respectively. After that, the normalized throughput of each batch is decreased as the number of vehicles increase. Maximum normalized throughput of Batch 1, Batch 2, and Batch 3 are 0.5848, 0.321 and 0.104, respectively.

**Figure 12:**
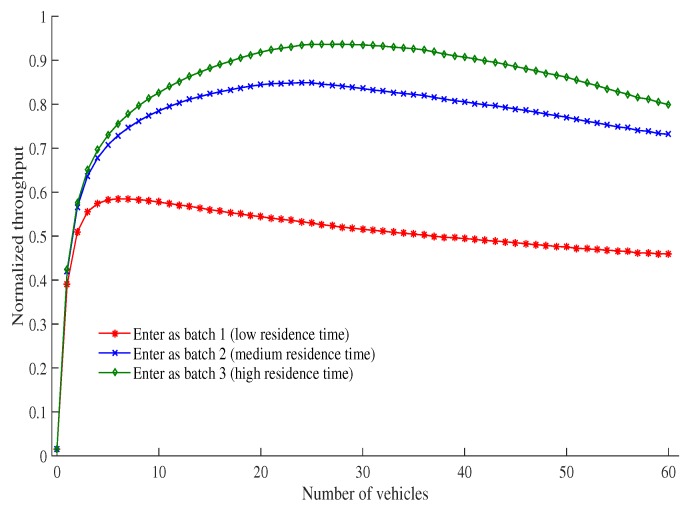
Total normalized throughput vs. number of vehicles.

Figure 12 shows the total normalized throughput of vehicles which are members of different batches at different times as the number of vehicles increase. We assume that initially three types of vehicles enter into the service area of an RSU with the velocities 5 ms^−1^, 9 ms^−1^ and 45 ms^−1^. These vehicle types keep their velocities throughout their entire residence time. According to Table 5, the first type of vehicles enter the network under Batch 3. The second type of vehicles enter the network as Batch 2, and the last type of vehicles enter the network under Batch 1. It can be observed from Figure 12 that the vehicles in Batch 3 have the longest residence time and achieve the highest normalized throughput because these vehicles get three times more opportunities to transmit data (as compared with the other two batches). Batch 2 has the second highest residence time, and has twice as many opportunities to transmit data (as compared with Batch 1). Finally, Batch 1 has the lowest residence time, and thus the lowest transmission normalized throughput. Our scheme assumes that the demand of transmitting data is proportional to the residence time of any given vehicle. It can be seen from Figure 12 that the total normalized throughput of vehicles is proportional to their residence time, and every vehicle (including Batch 1) has at least a minimum level of normalized throughput. Based on the definition of proportional fairness [15], this proposed protocol ensures proportional fairness. For example, the total normalized throughput of batches(for vehicle 30) which enter into the network as a member of Batch 1, Batch 2, and Batch 3 are 0.52, 0.84, and 0.94 when their maximum residence time are 11.11 s, 55.55 s and 100 s, respectively.

**Figure 13:**
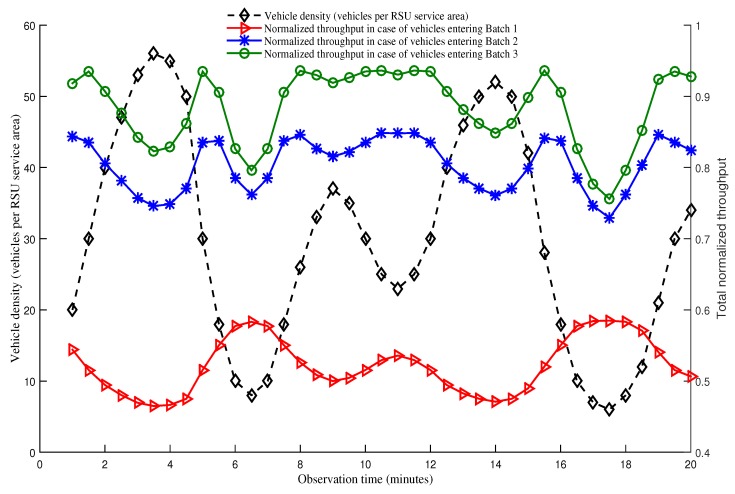
Relationship between vehicle density and total normalized throughput considering both saturated and non-saturated conditions.

Figure 13 shows the relationship between vehicle density, that is measured in terms of number of vehicles within a RSU service area, and total normalized throughput of vehicles belonging to different batches over a period of time. We assume that the observation period is 20 min, changing rate of vehicle density is equal for all batches, and the density of vehicles varies from 6 to 56. Y-axis at the left side represents the vehicle density whereas right-side Y-axis represents total normalized throughput of vehicles. There are three groups of vehicles in the network: First group of vehicles enter the network as a member of Batch 3. The second group of vehicles enter the network as Batch 2, and the last group of vehicles enter the network as Batch 1. It can be observed from Figure 13 that first group vehicles entering in Batch 3 have the longest residence time, and achieve the highest normalized throughput. The reason is that these vehicles get three times more opportunities to transmit data as compared to other two batches. Second group vehicles that enters in Batch 2 have the second highest residence time, and has as many as double opportunity to transmit data compared to that of Batch 1. Finally, vehicles in the last group that enters in Batch 1 have the lowest residence time, and thus have the lowest normalized throughput. Our protocol assumes that the demand of transmitting data is proportional to the residence time for any given vehicle. It is observed in the same figure that the total normalized throughput of vehicles is proportional to their residence time, scaled in right-side Y-axis. Moreover, the effect of density on total normalized throughput is observed by considering left-side and right-side Y-axis together. Figure 12 shows that the maximum normalized throughput (i.e., saturated condition) of vehicles which enter in the network as a member of Batch 1, Batch 2, and Batch 3 are achieved when the number of vehicles are 6, 24, and 28, respectively. Accordingly, this figure shows that the normalized throughput increases as vehicle density increases and vice versa in case of a non-saturation condition. However, when the network is in saturated condition, the normalized throughput decreases with the increase of vehicle density, and the other way around. For example, in this figure, it is observed that vehicles having high velocity and contending in Batch 1 sees the total normalized throughput being decreased as vehicle density increases and vice versa. This characteristics is observed because the total number of vehicles is always greater than 6, and the network goes to saturated condition for Batch 1. However, Batch 2 experiences both saturated and non-saturated condition. When number of vehicles of second group (i.e., entering as a member of Batch 2) is less than 24, the network is non-saturated which results in a throughput increase with the increase in density and vice versa. When the number of vehicles is greater than 24, opposite characteristics is observed as the network enters in saturated state. Vehicles in the third group also follow the same characteristic as group 2. However, this group enters in saturated condition when the number of vehicles reaches 28. Furthermore, it is also clearly observed that our proposed SAFE-MAC protocol always achieves proportional fairness in both saturated and non-saturated conditions for scenarios with a varying vehicle density.

**Figure 14:**
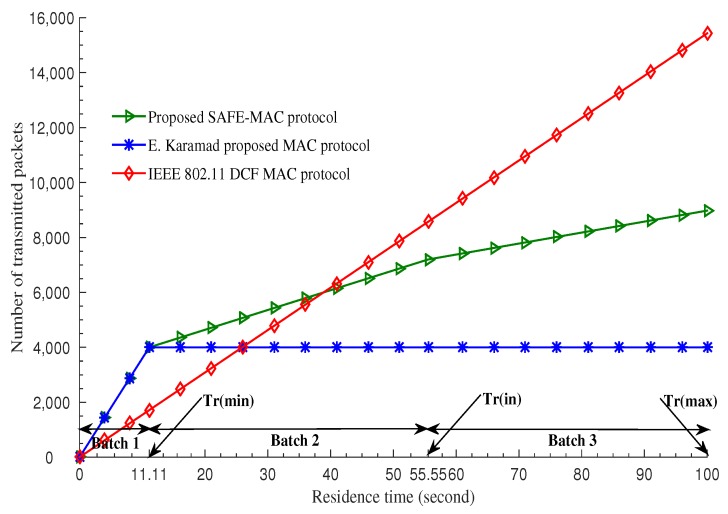
Number of transmitted packets vs. residence time [21].

Figure 14 shows the comparison of maximum number of transmitted packets of different MAC protocols with various velocities over residence time. IEEE 802.11 DCF MAC protocol [20] ensures that the number of packet transmitted is proportional to the residence time of the vehicles, but a minimum chance for all vehicles in not achieved. The E. Karamad protocol [3] ensures absolute fairness by ensuring the amount of packet transmission is constant regardless of velocity variation. Our protocol clearly demonstrates that the number of packets transmitted is directly proportional to the residence time and that a minimum channel access for high velocity vehicles is guaranteed. In this way, we have shown that our protocol meets the requirements for proportional fairness.

Finally, we present a comprehensive view of the comparisons in Table 6 that describes the percentage of transmitted packets of different batches under different MAC protocols. It can be seen that the IEEE 802.11 DCF MAC protocol [20], represented by the pie charts in the second column, ensures the number of packets transmitted be proportional to the total residence time of the vehicle as all the MAC parameters are commonly used by the vehicles. When the vehicle speed varies between 5 ms^−1^ and 35 ms^−1^, 58.3% packets are sent by the slowest moving vehicles that uses Batch 3 (B3). However, only 8.3% data can be sent by fast moving vehicles which contends in Batch 1 (B1). Vehicles with moderate speed that contend in Batch 2 (B2) send 33.3% data. As the variation in speed increases between the vehicles, the percentage of transmitted data becomes more unfair. For example, in case of the vehicles having speed between 5 and 85, Batch 1 (B1) gets only 3.7% share in the transmitted data while the slowest moving vehicles that contend in Batch 3 comprise 62% of the transmitted data. Therefore, the pie charts in the second column clearly show the unfairness issue of IEEE 802.11 DCF which gets more dominant in case of high variance in speed. E. Karamad proposed MAC protocol [3], represented in the third column, offers maximum transmitted data regardless of velocity variation and regardless of batch because each batch of vehicles use different MAC parameters constantly for whole residence time. This ensures that the total number of packets transmitted by each vehicles remains equal. Pie charts in column 3 show that all the batches i.e., B1, B2 and B3 send an equal percentage (33.3%) of data regardless of the variation in speed. As a result, the slowest moving vehicles cannot transmit more packets than the fast moving vehicles even though the residence time of the slowest moving vehicles is much higher than that of fast moving vehicles. Hence, E. Karamad protocol cannot provide enough opportunities to transmit packet for slow moving vehicles, thereby creating starvation problem. This protocol does not allow the amount of maximum transmitted data to be proportional to residence time. Finally, our proposed SAFE-MAC protocol, illustrated by the fourth column, offers an opportunity to transmit a minimum number of packets for every vehicle regardless of batch. At the same time, it also allows the amount of maximum transmitted data to be proportional to residence time. When the speed of the vehicles varies between 5 and 35, Batch 1 (B1), representing the fastest moving vehicles, can achieve a transmitted data ratio as high as 20.2% in contrast to only 8.3% in IEEE 802.11 DCF. If the speed variation increases further, for example vehicles having speed between 5 and 85, B1 can still achieve 19% whereas the highest percentage (44.9%) of transmitted data is achieved by Batch 3. This is because different batches use different MAC parameters, and vehicles are allowed to update their batch after a predefined time interval thereby allowing them to use the new MAC parameters (i.e., high priority batch). When velocity variation is increased, variation of maximum number of packets among the batches increases proportionally.

## 7. Conclusions & Future Work

In this paper, we designed a Speed Aware Fairness Enabled MAC protocol, SAFE-MAC, for V2I communications environments. It adapts the MAC parameters of each vehicle according to its residence time in the service area of a RSU to mitigate the fairness problem which arises due to the varying residence time of the vehicles. In the proposed scheme, small size minimum contention window is used by high speed vehicles or/and vehicles having small residence time. On the other hand, low speed vehicles or/and vehicles having high residence time use large size minimum contention windows. Since a small size minimum contention window provides more chance for communication thana large window, high speed vehicles get an optimal chance to transmit data with low speed vehicles. The proposed protocol ensures proportional fairness because each vehicle has a chance to adapt at least higher priority MAC parameters (e.g., small size minimum contention window). Furthermore, we have developed and applied an analytical model to test the performance of our proposed scheme in both saturated and non-saturated states. We also take into account all major factors that affect the access performance of our protocol, including the saturation condition, backoff counter freezing, channel idle, collision, successful transmission, packet drop and retransmission limit. Morover, we proposed two algorithms for residence time calculation and batch selection and update process. We derived the relationship between the transmission probability and minimum contention window size; we also derived the relationship between number of vehicles and some other important parameters including probability of collision, probability of packet drop, probability of channel idle, probability of successful transmission, probability of transmission and normalized throughput based on our analytical model. The effect of vehicle density on total normalized throughput in both saturated and non-saturated conditions is also investigated. Finally, we explore the relationship between residence time and maximum transmitted data of vehicles such that we could compare our performance against the basic access mechanism IEEE 802.11 DCF MAC and the E. Karamad MAC protocol. Accordingly, the proposed SAFE-MAC protocol overcomes the fairness problem by ensuring minimum access of all the vehicles for all applications in V2I communications Environment.

In the proposed SAFE-MAC protocol, we do not consider service differentiation to enhance the Quality of Service (QoS), as in IEEE 802.11p standard. Moreover, the fairness issue in case of Vehicle to Vehicle (V2V) and Vehicle to Device (V2D) communications is not considered here. Both these issues will be explored in our future work.

## Figures and Tables

**Table 1:** Batch selection and update.

Instantaneous Residence Time ($T_{r}$)	Batch No (Bi)	Maximum Residence Time ($T_{r(i)}$)	Priority (*i*)
0 <$T_{r}$ ≤ $T_{r(min)}$	B1	$T_{r(min)}$	High
$T_{r(min)}$ <$T_{r}$ ≤ $T_{r(in)}$	B2	$T_{r(in)}$	Medium
$T_{r(in)}$ <$T_{r}$ ≤ $T_{r(max)}$	B3	$T_{r(max)}$	Low

**Table 2:** Notations used in the model.

Notation	Definition
**$P_{i,j,k}$**	Probability of a vehicle of Batch *i*
	is in backoff stage *k* with backoff counter value *k*
**$P_{coll(i)}$**	Collision probability of Batch *i*
**$P_{idle(i)}$**	Channel idle probability when Batch *i* has a packet to transmit
**$P_{succ(i)}$**	Probability of successful transmission of Batch *i*
**$P_{drop(i)}$**	Packet drop probability of Batch *i*
**$P_{eqat(i)}$**	Probability that an vehicle of Batch *i* has empty queue after
	successful transmission or packet drop
**$P_{eq(i)}$**	Probability that an vehicle of Batch *i* has empty queue
**$W_{i,j}$**	Contention window size of Batch *i* at backoff stage *j*
**$CW_{min(i)}$**	Minimum contention window size of Batch *i*
**$CW_{max(i)}$**	Maximum contention window size of Batch *i*
**$m_{i}$**	Maximum stage of Batch *i* beyond which the
	contention window will not be increased
**$m_{i}+x_{i}$**	Retransmission limit of Batch *i*
**$P_{t(i)}$**	Transmission probability of Batch *i*
**$n_{i}$**	Average number of vehicle of Batch *i*
**$T_{succ}$**	Average time of a successful transmission
**$T_{coll}$**	Average time of a collision
**σ**	Duration of a empty CSMA slot
**$T_{p}$**	Average packet length in time
**$T_{i}$**	Average residence time of Batch *i*
**$S_{i}$**	Normalized throughput of Batch *i*
**$R_{i}$**	Packet transmission rate of Batch *i*

**Table 3:** System parameters.

Parameter	Value	Parameter	Value
$T_{p}\left(\mu s\right)$	8184	*H*	0.8
$T_{succ}\left(\mu s\right)$	8972	$R_{bit}\left(Mbps\right)$	1
$T_{coll}\left(\mu s\right)$	8713	$N_{bit}\left(byte\right)$	216
σ(μs)	50	D(m)	500
SIFS(μs)	28	$N_{P}$	4000
DIFS(μs)	128	$v_{max}\left(ms^{-1}\right)$	45
ACK(μs)	240	$v_{min}\left(ms^{-1}\right)$	5

**Table 4:** Parameters used in numerical calculations.

Parameters	Batch 1 (B1)	Batch 2 (B2)	Batch 3 (B3)
$CW_{min}$	3	9	30
$CW_{max}$	12	112	1280
$m_{i}$	2	4	7
$x_{i}$	4	2	0

**Table 5:** Batch selection.

Instantaneous Residence Time ($T_{r}$) (Sec.)	Batch No (Bi)
0 <$T_{r}$ ≤ 11.11	B1
11.11 <$T_{r}$ ≤ 55.55	B2
55.55 <$T_{r}$ ≤ 100	B3

**Table 6:**
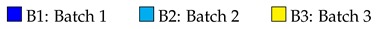
Performance comparison of SAFE-MAC with contemporary protocols in terms of percentage of transmitted packets while varying the difference between minimum speed and maximum speed.

	Standard IEEE 802.11 DCF MAC	E. Karamad & F. Ashtiani Proposed Protocol	Proposed SAFE-MAC Protocol
Speed varies between 5 and 35	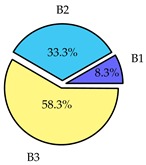	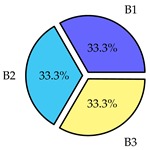	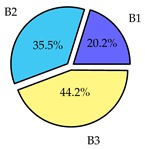
Speed varies between 5 and 45	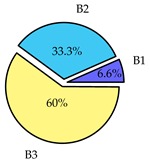	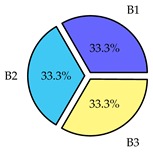	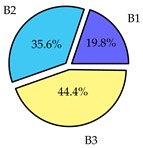
Speed varies between 5 and 65	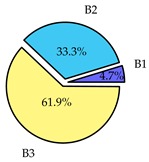	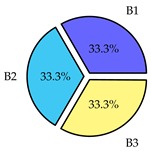	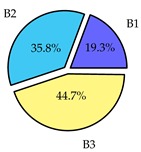
Speed varies between 5 and 85